# Metabolomics in NEC: An Updated Review

**DOI:** 10.3390/metabo14010014

**Published:** 2023-12-24

**Authors:** Alice Bosco, Claudia Piu, Marta Emanuela Picciau, Roberta Pintus, Vassilios Fanos, Angelica Dessì

**Affiliations:** Department of Surgical Sciences, University of Cagliari and Neonatal Intensive Care Unit, AOU Cagliari, 09124 Cagliari, Italy; alice.bosco@unica.it (A.B.); c.piu2@studenti.unica.it (C.P.); m.picciau2@studenti.unica.it (M.E.P.); roberta.pintus@unica.it (R.P.); vafanos@tiscali.it (V.F.)

**Keywords:** NEC, metabolomics, microbiomics

## Abstract

Necrotizing enterocolitis (NEC) represents the most common and lethal acute gastrointestinal emergency of newborns, mainly affecting those born prematurely. It can lead to severe long-term sequelae and the mortality rate is approximately 25%. Furthermore, the diagnosis is difficult, especially in the early stages, due to multifactorial pathogenesis and complex clinical pictures with mild and non-specific symptoms. In addition, the existing tests have poor diagnostic value. Thus, the scientific community has been focusing its attention on the identification of non-invasive biomarkers capable of prediction, early diagnosis and discriminating NEC from other intestinal diseases in order to intervene early and block the progression of the pathology. In this regard, the use of “omics” technologies, especially metabolomics and microbiomics, could be a fundamental synergistic strategy to study the pathophysiology of NEC. In addition, a deeper knowledge of the microbiota–host cross-talk can clarify the metabolic pathways potentially involved in the pathology, allowing for the identification of specific biomarkers. In this article, the authors analyze the state-of-the-art concerning the application of metabolomics and microbiota analysis to investigate this pathology and discuss the future possibility of the metabolomic fingerprint of patients for diagnostic purposes.

## 1. Introduction

Necrotizing enterocolitis (NEC) is the most common and lethal acute gastrointestinal emergency of newborns that mainly affects premature newborns, with an incidence of 5–12% in low-weight newborns (VLBW; <1500 g). This pathology is a necro-inflammatory lesion of the intestine with mortality rates of approximately 25% and serious long-term consequences [1,2,3]. In fact, intestinal resection is necessary in up to 40% of cases in premature newborns, and, in such circumstances, the mortality rate increases by up to 50%, together with significant subsequent morbidity, such as short bowel syndrome [3].

Its pathogenesis is complex and multifactorial, although today there is still no unanimous agreement on the importance of risk factors with uncertain causality [4,5,6]. Indeed, after a consensus in which 35 international NEC experts were asked to examine 64 identified risk factors, high agreement was only expressed for gestational age, birth weight and type of nutrition (milk, artificial or maternal) [5].

This was confirmed by the results of a more recent systematic review of the literature which highlighted the scarcity of high-quality prognostic studies on NEC and consequently a difficulty in establishing the predictive values of such risk factors [7]. Thus, there are maternal risk factors such as race, pre-eclampsia and premature rupture of membranes; birth-related factors such as the type of delivery or hypoxic/ischemic insults and, finally, post-natal ones such as infections and antibiotics administration, together with a dysregulation of the immune system and intestinal immaturity [3,4,8,9,10,11,12].

Moreover, the clinical picture of this pathology increases the degree of complexity, since, especially in the initial stages, it is characterized by mild and non-specific symptoms, often indistinguishable from those of sepsis, but which can progress towards significant and irreversible intestinal damage, up to a multiple organ failure and/or intestinal perforation. These characteristics strongly limit the therapeutic window [13].

Currently, the diagnosis of NEC is based on the combination of clinical, laboratory and radiographic findings, according to the modified Bell staging criteria [6,14]. However, these criteria are non-specific and cannot distinguish between NEC and non-NEC diseases. Indeed, some symptoms may be characteristic of other diseases, such as sepsis, and, based on these criteria, some spontaneous intestinal perforation can be misclassified as NEC. Moreover, these criteria include a number of unweighted characteristics based on the importance of the diagnosis of NEC [14,15,16,17]. 

Thus, the lack of specific clinical and radiological signs, combined with the poor diagnostic value of tests available nowadays, makes it difficult to achieve a sufficiently early diagnosis of NEC [15]. Therefore, for several years now, researchers’ efforts have focused on the identification of non-invasive biomarkers capable of prediction, early diagnosis and discriminating NEC from other intestinal diseases which can offer the opportunity for early intervention to block the progression of the pathology [6,18,19]. In this regard, to date, the use of “omics” technologies has expanded in the neonatal field for the complete and systematic detection of mediators capable of clarifying pathological mechanisms and host–pathogen interactions. Among these, metabolomics provides a real-time chemical profile of a biological system in terms of low molecular weight metabolites (metabolome) present in cells, tissues, organs and biological fluids, also allowing non-targeted investigations without a priori hypotheses conditioned by current knowledge and the results of previous research [20]. It also has further advantages compared to other omics approaches, as it represents the specific biochemical response of an individual to a stimulus, describing its phenotype. However, although a unifying metabolomic signature of NEC has not yet been identified, there is an increasing number of studies that have shown relevant evidence regarding NEC-related metabolic alterations inherent to particular pathways related to the inflammatory state, intestinal permeability and energy depletion. 

Moreover, the combination of this approach to microbiota analysis, with culture-independent high-throughput sequencing technologies, can allow us to better understand the microbiota–host cross-talk, also defining in more detail the possible pathogenetic contribution of the intestinal microbiota to NEC occurrence [20,21]. In fact, to date, although intestinal dysbiosis is recognized as an important pathogenetic contribution to NEC, the role of the intestinal microbiota still remains poorly defined [20,21].

## 2. Intestine and the Microbiota of Preterm Newborns 

The intestine of preterm newborns shows important differences compared to full-term newborns, being characterized first of all by an immaturity of the enterocytes which determines a reduced digestive and nutrient absorption capacity. It displays an alteration of the motility with consequent variation in the clearance of luminal contents and reduced production of mucus, caused by a lower number of goblet cells, which also determines less mechanical protection [22,23,24,25,26]. Other relevant characteristics of the intestine of the premature neonate are an altered microcirculatory perfusion, an immaturity of the immune system, an increased molecular expression and signaling activity of key mediators, such as toll-like-receptor-4 (TLR4), and a different bacterial colonization.
**HIGHLIGHT: PREMATURE GUT FEATURES**◆Enterocytes immaturity;◆Motility alteration;◆Lower number of goblet cells;◆Altered microcirculatory perfusion;◆Different bacterial colonization.

All these factors determine a more marked vulnerability of the epithelia, greater exposure to pathogenic bacteria and toxic luminal contents with consequent possible dysregulation of the inflammatory response [24,25,26,27]. As regards immune dysregulation and the altered inflammatory response, TLR4 and macrophages are of particular importance [23,24,28]. In fact, Hackman et al. [24,25,26] conducted numerous studies on the delicate role of TLR4 in the intestine of premature newborns, where this receptor is more expressed due to its important role in regulating the development of the intestinal epithelium during intrauterine growth. However, following the massive post-natal intestinal colonization, there is a significant activation of this receptor pathway by Gram-negative bacteria which determines a high basal level of stress on the endoplasmic reticulum of the enterocytes responsible for an increased risk of apoptosis which, in turn, can lesion the intestinal barrier, impair the repair systems and increase the production of pro-inflammatory cytokines. The latter factor appears closely related to the action of up-regulation of pro-inflammatory T helper 17 cells and the reduction in protective regulatory T cells, induced precisely by TLR4 hyper-activation. In addition, there could be a greater bacterial translocation due to the alteration of the permeability of the membrane, which can lead to the activation of TLR4 at the level of the mesenteric endothelium as well, with consequent reduction in blood flow and development of intestinal ischemia, necrosis and NEC.
**HIGHLIGHT: IMMUNE ALTERATIONS CHARACTERISTIC OF THE PREMATURE INFANT’S GUT**◆Increased molecular expression and signaling activity of key mediators (toll-like-receptor-4);◆Up-regulation of pro-inflammatory T helper 17 cells and reduction in protective regulatory T cells;◆Macrophage immaturity: highly pro-inflammatory profile and low phagocytic activity.

These pathogenetic mechanisms, defined as the “cross-switching hypothesis”, explain to a large extent not only the greater susceptibility of premature infants to NEC but also the role of intestinal bacterial colonization and the protective effect of breast milk [23,24,27,28]. In fact, 2′ fucosyllactose and 6′ sialyllactose, characteristic oligosaccharides of human milk, have the ability to bind directly to TLR4, inhibiting its signaling in cultured enterocytes, in enteroids derived from mouse intestine and in intestinal explants obtained at the time of surgical resection of patients with NEC [29]. A further contribution to NEC-related mucosal injury and intestinal barrier dysfunction, also mediated by the TLR4 signaling pathway, comes from platelet-activating factor [30]. It has in fact been observed that newborns with NEC have high circulating levels of platelet-activating factor and that this molecule induces the expression and signaling of TLR4 [30,31].

As regards intestinal macrophages, they express highly pro-inflammatory cytokines in premature newborns since the maturation process is completed only during gestational development, allowing them to acquire a non-inflammatory profile and good phagocytic activity [32]. However, under normal conditions, during intrauterine growth, the intestine is protected from such inflammatory responses thanks to transforming growth factor beta isoform 2 (TGF-β(2)). However, this protection is lacking during NEC. Indeed, there is a decrease in the tissue expression of TGF-β(2) and a lower bioactivity of TGF-β [32].

The microbiota of the preterm newborn is characterized by low bacterial heterogeneity with few anaerobes, higher levels of Enterobacteriaceae and potentially pathogenic bacteria such as Klebsiella pneumoniae and Clostridioides difficile [19,33,34,35,36,37], although interindividual variations remain high [36]. In addition, there is a delay in colonization due to beneficial microbes such as Bifidobacterium. Moreover, it has been hypothesized that the diversity of the composition and function of the intestinal microbiota of premature infants in the early postnatal period is strictly related to the biology of the host and is not justified by some typical characteristics of premature infants, such as the low rate of breastfeeding, antibiotics’ administration or caesarean section [34,38]. 

Indeed, Chu et al. [39] highlighted that within the first 6 weeks of life, the entire microbiota of the newborn undergoes a substantial reorganization, driven mainly by the body site and not by the mode of delivery. Therefore, the most relevant factor appears to be gestational age together with postnatal age, with the microbial population assembling more slowly in infants born more prematurely, supporting the fact that host biology is the main driver of bacterial colonization in the intestine of premature newborns [36,38]. Then, Rosa et al. [38] highlighted that the gut microbiota of premature infants residing in a tightly controlled microbial environment (a microbiologically constrained ecosphere of an intensive care unit) progresses through a succession of bacterial classes from Bacilli to Gammaproteobacteria to Clostridia, interrupted by abrupt population changes, with good colonization by anaerobes only around the postconceptional age of 33–36 weeks (corresponding to the third–twelfth week of life depending on the gestational age at birth). Furthermore, they highlighted that external factors, such as antibiotics, vaginal or cesarean delivery, the type of breastfeeding and the age of the newborns at the time of sampling, influence only the pace but not the sequence of this progression. In fact, they found that breast milk appears to be associated with a greater proportion of Gammaproteobacteria limited to 28 weeks of gestation. However, there are numerous studies that have highlighted not only a more significant influence of the timing and type of exposure to antibiotics, both intrapartum [36,40] and postnatal [36,41,42], with the most important repercussions on the Bifidobacteria, but above all the type of feeding, with important long-term effects [37,43,44,45,46]. 

In this regard, Arboleya et al. [37] conducted the first study aimed at evaluating the intestinal bifidobacterial composition at the species level in premature infants fed with human milk from donor mothers. From the analysis of 42 premature infants fed with human donor milk (DHM) vs. breast milk, a specific bifidobacterial profile based on the type of feeding was highlighted, with greater bifidobacterial diversity in the DHM group. These results reflect the development of the so-called “milk-oriented microbiota” with positive and long-lasting effects typical of breast milk that appear to be determined by the evolution of the bifidobacterial microbiota. Indeed, at two days of life alpha diversity was not distinct between groups, but both the diversity and the number of species detected decreased over time in the MOM group, which was probably selected based on the infant’s feeding mode. Finally, in support of the important protective role of breast milk, both expressed and donated, against some microbiota-related pathologies, typical of these newborns, such as NEC, there is a Cochrane Systematic Review and meta-analysis that was conducted in 2019 by Quigley et al. [47] and a systematic review and meta-analysis conducted by Altobelli et al. [48] in 2020. These data support the important role of breast milk not only in the correct maturation of the microbiota but also in its strong impact on the maturation of the intestinal barrier thanks to the presence of numerous growth factors, protection from infections due to the presence of antimicrobial factors, such as lactoferrin and lysozyme, and secretory IgA, which could bind opportunistic or pathogenic bacteria in an antigen-specific and non-specific manner [49,50,51,52].

## 3. The Microbiota in Necrotizing Enterocolitis 

**Figure 1:**
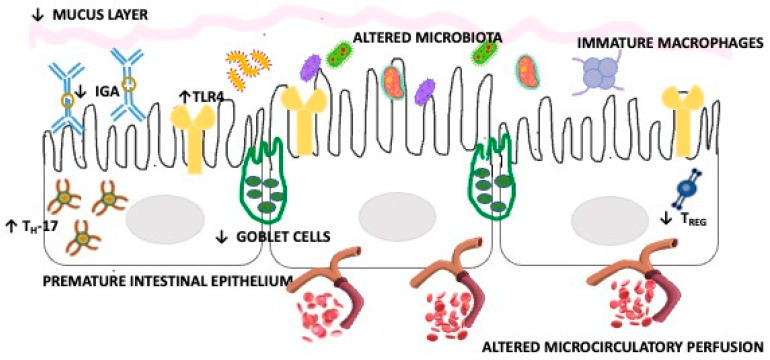
Premature gut characteristics promoting NEC onset. Abbreviations: ↓decrease, ↑ increase.

The alterations in the intestinal microbiota seem to play a key role in the genesis of NEC in the context of altered maturation characteristics of the intestine of premature infants, as shown in Figure 1.

In fact, animal models have shown that this pathology cannot be reproduced in germ-free animals [45]. Furthermore, the association between early antibiotic administration and NEC further supports the role of intestinal dysbiosis, which has also been related to immune system dysregulation, especially the already discussed TLR4 hyper-activation by LPS [53]. Recently, the overcoming of the use of culture techniques in favor of more advanced technologies based on the study of the microbial genome and metabolome has allowed us to expand knowledge regarding the role of dysbiosis as a possible cause of NEC, compared to a single pathogen [54]. In fact, nearly 90% of microbial species cannot be easily cultured with current laboratory culture techniques. The most common sequencing approach to analyze the microbiome is the analysis of amplicons of the 16S ribosomal RNA (16S rRNA) gene, a part of the ribosomal RNA, and its sequencing by PCR with primers that recognize highly conserved regions of the gene so that we can compare the composition of the microbiota between samples. However, this technique also has some limitations since it is based on the putative association of the 16S rRNA gene with a taxa, defined as an operational taxonomic unit (OTU), analyzed at the phyla or genera level and therefore less precise at the species level. Then, there is whole-genome shotgun sequencing (WGS), which uses random primer sequencing to sequence overlapping regions of a genome, allowing precise identification of taxa at the species level [54].

Studies conducted on NEC-related dysbiosis, especially in preterm infants, have increased in recent years. Pammi et al. [53], in order to distinguish and analyze intestinal dysbiosis before the onset of NEC, conducted a systematic review and meta-analysis of microbiome profiles in preterm infants. They included studies that compared the gut microbiome of preterm infants who went on to develop NEC with that of controls, using culture-independent molecular techniques. The results highlighted that the fecal microbiome of preterm infants before the development of NEC was characterized by an increase in the relative abundances of Proteobacteria and a decrease in the relative abundances of Firmicutes and Bacteroidetes. In fact, although the microbial profiles highlighted by the 14 studies included in the review were variable, 8 showed an increase in the phylum Proteobacteria in the feces of newborns who developed NEC. Furthermore, it emerged that both the alpha and beta diversity index in preterm infants with NEC were not different from controls, while taxonomic differences were highlighted in relation to exposure to antibiotics, artificial feeding and mode of delivery. The association between Proteobacteria and NEC strengthens the hypothesis of an exuberant response of the organism to the products generated by Gram-negative bacteria and the consequent activation of TRL4.

Dobbler et al. [55] showed similar results in stool samples collected from the first day of life (including meconium) up to the fifth week (or diagnosis of NEC) of 40 preterm newborns, including 29 controls and 11 NEC cases, through a 16S rRNA analysis. They highlighted, in cases of NEC, a lower microbial diversity and an anomalous development of the microbial community before the diagnosis of NEC, with an increase in Proteobacteria from the second to the third week. Differently, the controls were characterized by a constant increase in Firmicutes and a decrease in Proteobacteria, Bacteroidetes and Actinobacteria. The presence of a single phylotype that is closest to the Enterobacteriaceae family, which has proven to be strongly related to NEC, has also emerged. Through a shotgun metagenomic sequencing of the DNA of the sample with the highest abundance of this phylotype, the presence of *Citrobacter koseri* and *Klebsiella pneumoniae* as dominant taxa was highlighted. The authors therefore hypothesized that these two taxa could represent suitable microbial targets for the early diagnosis of NEC.

A large-scale analysis of the intestinal microbiome (47 datasets from the study and 124 retrieved from public archives) with a shotgun sequencing technique was conducted by Tarracchini et al. [21] in order to analyze the intestinal microbiota of preterm infants. They highlighted both the differences in the microbiota between newborns affected by NEC and before the development of the pathology. Their meta-analysis outlined the presence of precise typologies of recurrent preterm community states (PT-CST) in controls and NEC newborns. Specifically, excessive growth of a number of opportunistic microbial species together with a loss of intestinal microbial biodiversity has been observed in cases of NEC. Indeed, the index of bacterial species richness (biodiversity) was on average relatively lower than that of full-term newborns, although statistically higher in healthy subjects compared to NEC subjects in which the two most abundant microbial species in fecal samples cover 38% of the entire bacterial population, while those of healthy newborns cover only 29.76%. This led the authors to hypothesize that a decrease in even a few species can markedly alter the delicate ecological balance established in the very early stages of life of the human intestinal environment. Furthermore, the analysis of the inter-sample variability of the composition of the intestinal microbiota revealed significant differences between NEC infants and control samples supporting that the intestinal microbiome of NEC infants shows not only a lower biodiversity but also a taxonomic composition different from that of their counterparts. In addition, thanks to a longitudinal analysis of preterm newborns before the development of NEC, the presence of *Clostridium neonatale* and *Clostridium perfringens* was highlighted as a potential early indicator of the development of this disease. Furthermore, the shotgun metagenomics data were also used to investigate the presence of alterations in particular metabolic pathways, revealing that the enzymes involved in the metabolism of tryptophan in the synthesis of biotin and the degradation of human milk oligosaccharides were depleted in the samples of preterm infants diagnosed with NEC compared to controls. Moreover, the enzyme lactate dehydrogenase (LDH) was overabundant in preterm infants before the development of NEC. These data led the authors to hypothesize a possible gastrointestinal accumulation of DL-lactate in newborns who then develop NEC, supporting the potential use of this compound as a potential functional biomarker for the early diagnosis of NEC. In detail, some enzymes linked to the degradation of glycosylated proteins, such as a-fucosidase and sialidase, which are characteristic of healthy newborns, were almost completely absent in NEC cases. These enzymatic pathways normally reflect the presence of a healthy microbiota characterized by *Bifidobacterium longum*, *Bifidobacterium brevis* and *Bifidobacterium bifidum.* In NEC subjects, however, values three times higher of key enzymes in the tryptophan degradation pathways, such as tryptophanase and indole-pyruvate decarboxylase, were highlighted. From the positive correlation index, the authors hypothesized that these values may be the result of a greater abundance of *E. coli* in NEC subjects, as well as members of the Klebsiella and Staphylococcus genera. On the contrary, in controls, a fourfold increase in the enzyme tryptophan 2,3-dioxygenase (TDO) was detected, which catalyzes the first phase of the kynurenine pathway, an alternative pathway of tryptophan metabolism responsible for the production of metabolites with neuroactive properties that also regulate various biological processes linked to inflammation and the immune response. These data led the authors to hypothesize that the depletion of bacteria homologous for the TDO enzyme (typically a eukaryotic enzyme that some bacteria can synthesize thanks to a homologous enzyme) in NEC subjects may reflect increased intestinal inflammation and/or an unbalanced reactivity of the intestinal mucosa. Indeed, positive associations emerged between the abundance of TDO and the presence of various commensals of the infant gut, including the genera Bacteroides, Cutibacterium and Enterobacter, while negative correlations were identified with *E. coli* and *S. epidermidis.* Other metabolic differences emerged for some enzymes involved in various catabolic and anabolic pathways in humans, with overexpression in NEC fecal samples of the glycogen debranching enzyme, indicated as a potential virulence factor in many microorganisms, and a decreased expression of enzymes linked to the metabolism of biotin, a vitamin that can be synthesized at the intestinal level by some intestinal commensal bacteria characteristic of a healthy microbiota, such as *Bacteroides fragilis*. Furthermore, a negative correlation coefficient was highlighted between the components of the biotin biosynthetic pathway and *E. coli*.

A further contribution regarding the role of the Clostridia class in NEC had already emerged from the prospective case–control study conducted by Zhou et al. [56]. They observed that, close to the onset of NEC, the abundances of Clostridium *sensu stricto* of the Clostridia class were significantly higher in subjects with early-onset NEC compared to controls. Differently, in late-onset NEC, Escherichia/Shigella among Gammaproteobacteria showed an increasing trend before disease onset and were significantly higher in cases than in controls six days before NEC onset. The authors therefore hypothesized that the specific infectious agent associated with NEC may vary based on the age of the host at disease onset, with greater differences in the microbiota in cases of early-onset NEC.

Ward et al. [57] conducted a large-scale shotgun metagenomic sequence analysis of the early gut microbiome of 144 preterm and 22 full-term infants. Through a pan-genomic approach to the functionally subtype of *E. coli*, they identified genes associated with NEC and mortality that indicate colonization by uropathogenic *E. coli* (UPEC). Multi-locus sequence typing metagenomic analysis further defined NEC-associated strains as sequence types often related to urinary tract infections, including ST69, ST73, ST95, ST127, ST131 and ST14. Therefore, UPEC colonization has been identified as a significant risk factor for the development of NEC and subsequent mortality.

From a clinical and microbiological point of view, most studies on NEC have focused only on established and severe phenotypes, such as NEC-2 and NEC-3, while suspected NEC (NEC-1) are still little studied. NEC-1 is characterized by lethargy, bradycardia, thermal instability associated with residual gastric bile, vomiting, abdominal distension with or without rectal bleeding with a normal abdominal radiographic image or simple dilatation [57].

Brehin et al. [58] analyzed the intestinal microbiota, microbiome and metabolome of children with suspected NEC-1. The results of this research highlighted that the intestinal microbiota of NEC-1 children had a greater abundance of Streptococcus species (from the 10th to 20th day of life) and Staphylococcus (from the 20th to 30th day of life), with the most significant evolutionary differences, also from a metabolomic point of view, found especially from the 20th to the 30th day of life. The NEC-1 microbiome was also more sensitive to childbirth, low birth weight and gestational age than the healthy microbiome. This led to a hypothesis of a precise time window (from the 20th to the 30th day of life) useful to prevent the progression of NEC. In fact, through the analysis for ten-day periods it was possible to detect a divergence for both the intestinal microbiota and the microbiome in NEC-1 within the period from the 20th to the 30th day of life, especially due to the greater abundance of Staphylococcus, suggesting that these days of life are an optimal time window for the administration of antibiotics against the bacterial species most present in NEC-1. Furthermore, given that NEC-1 infants subjected to glycopeptide and aminoglycoside therapy were more numerous than healthy children, the authors hypothesized that NEC-1 may be associated with resistance to glycopeptides and/or aminoglycosides, since the intestinal microbiota in NEC-1 was characterized by an increase, not a decrease, in Staphylococcus. In addition, given the activity of aminoglycosides against enterobacteria, their administration could delay intestinal colonization by Proteobacteria, thus favoring the implantation of resistant genera, such as Staphylococcus and Streptococcus. This led the authors to conclude that it would be useful not to prolong antibiotic therapy beyond the first week of life in preterm newborns if the inflammation indices have normalized. The study conducted by McMurtry et al. [59] also compared the intestinal microbiota of newborns with NEC classified into three subgroups based on severity (mild, severe and lethal) with that of healthy controls. The results did not highlight a specific microbial community consistently associated with NEC, with significantly lower bacterial diversity and relative abundance of Actinobacteria and Clostridia in NEC samples compared to controls. In addition, it was highlighted that microbial diversity together with the abundance and prevalence of Clostridia decreased with increasing severity of NEC. The differences between cases and controls, and those between the three subgroups, suggest how the microbiota can provide information both for diagnostic purposes and with regard to the severity of the disease. The progressive reduction in Clostridia with the increase in the severity of the pathology in the cases made the authors hypothesize a possible role in attenuating the inflammation leading to NEC for the Clostridia taxa, although other studies have demonstrated a different trend [20,21,56]. Furthermore, in this study, unlike others discussed previously, not only there was no difference found between the previous administration of antibiotics by the NEC and control groups but also no difference in Enterobacter abundances was observed between NEC and controls.

## 4. Supporting Early Diagnosis of NEC: Potential Biomarkers 

Given the limitations of clinical and radiological findings in the early diagnosis of NEC, in an attempt to predict, diagnose early and discriminate NEC from non-NEC intestinal diseases, the scientific community has focused on the discovery of new biomarkers [6]. However, the lack of a standard and well-agreed definition of NEC also undermines the research and discovery of effective biomarkers. In fact, the performance of biomarkers (sensitivity, specificity, likelihood ratio) must be sufficiently good to be useful without causing undesirable consequences due to false positives or negative NEC cases [60].

In their review, Agakidou et al. [6] state that the biomarkers discovered to date can be classified into three categories: the first includes hematological indices, such as the total white blood cell count, the absolute neutrophil count, the ratio between immature white blood cells and total white blood cells (I:T ratio) and platelet count. The second includes acute phase reactants, while the last includes immunological markers, including cytokines, chemokines, adhesion molecules, intracellular signal transduction molecules and growth factors. Nonetheless, the poor specificity of these biomarkers does not differentiate NEC from other inflammatory diseases of newborns. Furthermore, their diagnostic accuracy varies depending on the severity of NEC and the time of the disease course.

Recently, other more specific and potentially promising biomarkers for NEC have been investigated. Among these, the most evaluated for the prediction and diagnosis of NEC are fecal calprotectin (CP), the intestinal fatty acid binding protein (I-FABP), claudins and Trefoil factor 3 (TFF3). However, the studies conducted so far did not detect a reliable early biomarker, although the existing data overall suggest that I-FABP is a promising biomarker for NEC with the potential to improve its diagnostic value when combined with other markers of intestinal damage such as TFF3. In fact, although TFF3 was not specific for NEC, in combination with I-FABP, it may represent an early predictor of the outcome of NEC [6].

Another area of investigation regarding the detection of early biomarkers for the diagnosis of NEC is the analysis of compounds in breathing air. In fact, in the past century, two research groups [61,62] highlighted the potential role of exhaled hydrogen (H2) as an early biomarker of NEC. They had shown that high excretion of H2, potentially related to gut dysbiosis, preceded the first clinical signs of NEC by 8 to 28 h. This led the authors to hypothesize that exhaled H2 testing could be a simple, noninvasive test useful in the management of premature infants at risk of developing NEC [61,62]. 

Based on these findings, it was subsequently hypothesized that the overproduction of bacterial fermentation byproducts, such as short-chain fatty acids (SCFAs), may exert a causal role in the pathology [63]. The importance of this field of investigation lies not only in the fact that it is directly related to gut dysbiosis, a key feature in the pathogenesis of NEC, but also because of its evolution in the analysis of fecal volatile organic compounds (VOCs), which will be discussed in the following paragraphs.

## 5. NEC Metabolomics Biomarkers

**Figure 2:**
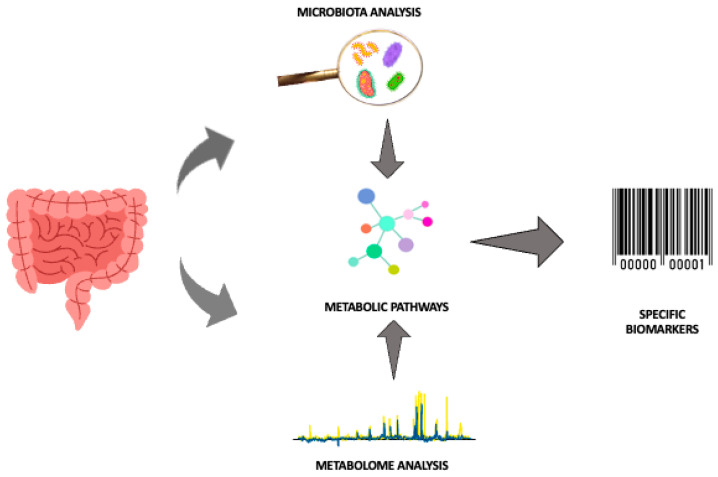
Combination of microbiota analysis and metabolomics in premature gut for NEC diagnosis optimization. Abbreviations: ↓decrease, ↑ increase.

In this context, “omics” technologies may be useful, especially untargeted metabolomics, which is not conditioned by current knowledge and the results of previous research, allowing the metabolites in a biological fluid to be comprehensively investigated, without a priori hypotheses [64]. Furthermore, modern analytical technologies allow the identification of patterns that provide much more information than measuring a single parameter. In fact, metabolomics, an innovative analytical profiling technique, allows the detection of the complete set of low molecular weight molecules (including sugars, lipids, small peptides, vitamins and amino acids) present in cells, tissues, organs and biological fluids, making it possible to evaluate specific and individual metabolic responses to different stimuli, highlighting a specific phenotype. This can lead to a possible identification of specific biomarkers with high diagnostic power thanks to the rapid and dynamic detection of changes in the concentration of metabolites through minimally invasive sampling, such as the collection of biological fluids such as blood, saliva, urine and feces [64]. Thus, the high volume of data generated in association with new technologies for analyzing the intestinal microbiota can significantly improve the knowledge of microbiota–host cross-talk by providing new information on the role of early nutrition, pharmacological therapies and immunomodulation in newborns [20]. Indeed, the intestinal microbiota exerts a broad effect on host biochemistry through the production of secondary metabolites. Furthermore, although the metabolites production is specific for each individual, the metabolic pathways in which they are involved, are the same. Thus, different bacteria can produce or modify common metabolites at different levels [65]. Therefore, thanks to the analysis of metabolites we can better understand how the composition of the microbial community can be altered, subsequently affecting the health of the host [50]. The potential synergy between microbiota analysis and metabolomics for the search for specific biomarkers in order to optimize the diagnosis of NEC is summarized in Figure 2.

We performed a literature review using NEC and metabolomics as keywords, in order to assess the state-of-the-art application of this technique in the investigation of this pathology starting from one of the first publications on the subject that was conducted by our research group in 2016 [19]. Only research articles with electronically available English full text were taken into consideration. 

Despite the progress of omics technologies in the last decade, to date, the number of studies examining metabolomics in infants with NEC is still quite small, as shown in Table 1. Indeed, the search yielded 17 articles, and among these, only 5 papers included microbiota analysis.

The first study regarding urinary metabolomics and microbial analysis in NEC was performed by Morrow et al. [66] in 2013. They performed both 16S rRNA gene sequencing analysis of stool samples and urinary metabolomic characterization of less than 29 weeks of gestational age infants, including 11 infants who developed NEC and 21 matched controls without NEC. The sampling was conducted before the onset of the disease highlighting two distinct forms of intestinal microbial alteration that preceded all cases of NEC, with repercussions on the metabolomic profile, revealing a strong correlation between NEC and dysbiosis. Indeed, although analysis of urine samples from days four to nine did not detect metabolites associated with all cases of NEC, further analysis showed that changes in individual urine amino acids were associated with the microbiome shifts that precede the development of NEC. Specifically, alanine and histidine significantly distinguished Bell stage I NEC from stage II NEC, with alanine being positively associated with cases of NEC preceded by Firmicutes dysbiosis, all NEC I, and histidine inversely associated with cases of NEC preceded by Proteobacterial dysbiosis, all classified as NEC II. Nonetheless, none of these amino acids alone was significantly associated with the overall risk of NEC while a high urinary alanine:histidine ratio provided a good prediction of overall NEC, making it a potential biomarker.

Another study on the urinary metabolome was performed by Thomaidou et al. [15]. They analyzed urine samples on the day of initial diagnosis of NEC from 15 preterm newborns with NEC (five Bell Stage I and ten Stage II/III) and from 15 controls, with untargeted nuclear magnetic resonance spectroscopy and targeted liquid chromatography with tandem mass spectrometry. Multivariate statistical models with data from both analytical approaches showed a clear separation between the metabolic profiles of neonates with NEC and controls, highlighting 25 discriminating metabolites belonging to amino acids and organic acids, sugars and vitamins. Some combinations of metabolites have shown excellent diagnostic performances in identifying newborns who develop NEC. In particular, they detected a reduction in some amino acids such as alanine, asparagine, tyrosine and proline in cases compared to controls. The reduced levels of alanine could be attributable to its increased degradation in the Krebs cycle, or related to intestinal dysbiosis. At the same time, the reductions in asparagine and proline, important for intestinal integrity and as a source of energy for enterocytes, could be associated with their increased consumption in the Krebs cycle by intestinal cells. On the contrary, some indicators of protein degradation such as arginine and phenylalanine were increased in cases of NEC. The increase in the latter can be correlated to the reduction in tyrosine levels, which according to the authors can derive, in children with septicemia, from an altered balance between phenylalanine retention, hydroxylation and tyrosine homeostasis due to the state of stress. Another discriminating metabolite is betaine, a protective osmolyte, which is decreased in cases of NEC compared to controls. The decreases in citrate and fumarate, two intermediates of the Krebs cycle, probably indicate energy depletion due to increased demands in inflammatory states. Significantly reduced concentrations of polyols such as mannitol/sorbitol, arabitol/xylitol and xylose were also detected in neonates with NEC compared to controls, a condition potentially related to the regeneration of reduced glutathione via the pentose phosphate pathway. Vitamins were also altered in subjects with NEC, with a decrease in riboflavin and an increase in pyridoxine. However, despite the results being promising, they need to be validated in larger studies.

The latest study on the urinary metabolome in NEC was recently performed by Picaud et al. [76]. In order to study changes in the urine metabolome of very low birth weight preterm infants with NEC and feeding intolerance, they conducted a longitudinal study in the first two months of life. Prospectively recruited preterm VLBW newborns’ urine samples were analyzed, six with NEC and food intolerance (Group 1, NEC), six with food intolerance without any sign of NEC (Group 2, FI) and six with good digestive tolerance without NEC (Group 3, GDT). Urine samples from NEC cases were collected and analyzed before, during and up to two months after the disease, while in controls, samples were collected as close as possible to the postnatal age of infants with NEC. The metabolomic analysis was conducted with proton nuclear magnetic resonance spectroscopy (H^1^ NMR). The results showed that in all preterm infants, urinary levels of betaine, glycine, succinate and citrate correlated positively with postnatal age. As regards suberate and lactate, the correlation with postnatal age emerged only in preterm infants with NEC and in controls with food intolerance. Moreover, N,N-dimethylglycine (N,N-DMG) correlated only in controls with good digestive tolerance. It also emerged that in preterm controls with food intolerance, there was a significant progressive decrease in N-methylnicotinamide and carnitine. Furthermore, lactate, betaine, myo-inositol, urea, creatinine and N,N-dimethylglycine discriminated late-onset NEC from controls with good food tolerance, confirming previous results associating the reduction in urinary betaine with NEC [15]. These results confirm that the urinary metabolome of preterm infants with NEC differs significantly from that of premature infants with food intolerance without NEC and from that of premature infants with good digestive tolerance.

In summary, data from the urinary metabolome in NEC seem to confirm its potential predictive role, showing alterations in urinary amino acid composition, potentially related to alteration of the gut microbiota and some metabolites such as betaine.

In 2015, in an exploratory non-targeted study, Wilcock et al. [67] tried to identify the presence of early serum metabolome biomarkers of NEC. A total of 2 serum samples A and B (“A”: at ≤1 week of age and “B”: once fully fed) from 12 preterm infants (less than 30 weeks of gestation) of whom 7 developed NEC and 8 term controls were analyzed. Metabolomic differences were found in preterm infants at risk of NEC only when full enteral feeding was introduced, part of which related to the up-regulation of IL-1β. Indeed, the network analysis highlighted an upregulation of IL-1 β before the clinical diagnosis of NEC. However, the sample size was not sufficient to identify a biomarker. 

Stewart et al. [69] also focused on finding serum biomarkers of NEC. They conducted a longitudinal analysis of the metabolome and serum proteome in preterm infants with NEC (six cases) or late-onset sepsis (LOS, four cases), matched with nine controls. The sampling was performed 14 days before diagnosis, at the time of the diagnosis itself and after the diagnosis. The results led the authors to conclude that it is unlikely that there is a single biomarker for NEC and/or LOS. In fact, in all cases of NEC or LOS, no single protein or metabolite was detected that was absent in controls although several disease state-associated proteins, potentially related to the different pathophysiology of the disease, were identified. 

At the same time, Silvester et al. [71] conducted a metabolomics study in dried blood samples collected by heel prick. They performed a retrospective cohort study to analyze the association between newborn acylcarnitine profiles and the subsequent development of NEC, using neonatal screening data routinely collected in preterm newborns. A model development cohort of 94 110 preterm births was used to develop a risk stratification model which was then applied to a validation cohort of 22 992 births. In these samples, the abnormality of fatty acid metabolism has been associated with prematurity and the development of NEC. In fact, the levels and ratios of as many as 14 acyl-carnitines have been associated with an increased risk of developing NEC. Furthermore, 6 acyl-carnitines values together with birth weight and total parenteral nutrition identified 89.8% of neonates with NEC in the model development cohort and 90.8% of neonates with NEC in the validation cohort.

Then, Sinclair et al. [75], to analyze the relationship between nutrition, metabolites and NEC, conducted a multicenter longitudinal study of blood samples from 887 preterm newborns (<32 weeks of gestation), of which 73 were cases of NEC, taken on days 1, 7, 28 and 42 of life. A panel of amino acids and acyl-carnitines was analyzed by tandem MS, which highlighted how early levels and serial variations of a series of metabolites were associated with the development of NEC. Indeed, at day 1, levels of alanine, phenylalanine, free carnitine, C16, arginine, C14:1/C16 and citrulline/phenylalanine were associated with subsequent development of NEC. These alterations in the levels of individual NEC-related metabolites varied over time with a predominance of amino acids at birth to a predominance of acyl-carnitines at day 42. Furthermore, the longitudinal model highlighted that several individual metabolites or metabolite ratios show significant associations with NEC. Specifically, a decrease in 3-hydroxyoleylcarnitine and the citrulline/phenylalanine ratio was observed while the phenylalanine/tyrosine and octanoyl-carnitine/decanoyl-carnitine ratios were up-regulated in the NEC cohort compared to controls. It was also highlighted that the subjects who developed NEC received fewer total calories (adjusted for significantly lower weight), a trend that emerged within the 7th day of life and which persisted until the 28th day of life and up to the 42nd day. The authors therefore concluded that NEC infants are characterized by metabolic abnormalities that progress in parallel with significant differences in nutritional intake, supporting a possible metabolic dysfunction in preterm infants before the onset of the disease. However, the effect of nutritional intake on the metabolic changes observed in this study needs to be further clarified.

In 2022, Thomaidou et al. [77] performed a case–control prospective study to investigate the blood metabolic profiles of NEC and LOS newborns. Blood samples from 15 septic newborns and 17 newborns with NEC were analyzed upon clinical suspicion of the specific pathology compared to those of 16 control newborns with corresponding gestational and postnatal ages without sepsis or NEC. Metabolomics analysis revealed significant differences in the metabolic profile of newborns with LOS or NEC compared to controls. Specifically, some differences in the metabolome have been isolated in newborns with NEC. Indeed, L-carnitine discriminated infants with NEC from those without NEC, while 3 glycerophospholipids, PC (16:0/0:0) or LysoPC (16:0/0:0) and PC (18: 1/0:0) or LysoPC (18:1/0:0) were significantly decreased and PC (20:4/0:0) significantly increased in cases. 

Overall, studies conducted on the blood metabolome also showed good predictive potential. In particular, the blood metabolome showed that the main alterations involved fatty acid metabolism (acyl-carnitines), but amino acid changes similar to what was observed in the urinary metabolome were also highlighted. The alanine alterations can be due both to the aforementioned dysbiosis and to a general alteration in metabolism, and, with regard to phenylalanine alterations, may be due to an alteration in protein degradation.

Other metabolomics studies were conducted on fecal samples of infants with NEC. Stewart et al. [70] analyzed 7 newborns who developed NEC and 28 matched controls from a cohort of 318 newborns, with gestational age less than 32 weeks. This study highlighted the need for a systems biology approach, at least based on metabolomics and microbiomics, in the identification of robust biomarkers for NEC with a view to an early and accurate diagnosis of the disease. In fact, a detailed temporal bacterial and metabolomic profiling of the intestinal microbiome was conducted during the disease, which highlighted a central community of Klebsiella, Escherichia, Staphylococcus and Enterococcus in all samples. The gut microbiota profiles clustered into six distinct clusters, termed preterm gut community types (PGCTs), each reflecting the dominance of major operational taxonomic units (OTUs), except PGCT 6, which was characterized by high diversity with Bifidobacteria dominance. It was found that NEC infants had significantly more PGCT transitions in clusters one to five before diagnosis, supporting the poor uniformity of the microbial signature in NEC, while PGCT 6 was found only in healthy samples. However, although no specific microbial signature was found before NEC diagnosis, metabolomic profiling showed the presence of five discriminating metabolites in the samples. These metabolites corresponded to two metabolites from the pathways of C21-steroid hormone biosynthesis and linoleate metabolism, two metabolites exclusive to linoleate metabolism, and one metabolite from the pathway of leukotriene metabolism and prostaglandin formation from arachidonate. The greatest significance of these metabolites was found at the time of diagnosis, with significantly higher values compared to all control samples, but their intensity increased before diagnosis and reduced after NEC diagnosis. These trends led the authors to assume that these molecules have a good predictive potential for the onset of the disease. If this is confirmed in larger studies, there could be the possibility of anticipating the diagnosis by 1–2 weeks compared to the current clinical diagnosis. Furthermore, a comparison of metabolomics data with each PGCT highlighted clear concordance with 16S bacterial profiling data, with the features most associated with NEC reduced in relative abundance in PGCT 6.

Wandro et al. [72] performed a stool metabolomics and microbiomics analysis, as well. They characterized the bacterial composition by 16S rRNA gene sequencing and the metabolomes by untargeted gas chromatography-mass spectrometry of 77 fecal samples from 32 very low birth weight preterm infants in the first 6 weeks of life. Unlike other studies, fecal microbiome and metabolome were found to be unique for each individual, emphasizing a personalized approach. In addition, no measured metabolites were associated with necrotizing enterocolitis, late sepsis, or positive outcome. The authors therefore concluded that, overall, both the microbiome of preterm infants and the metabolome were personalized and that the gut microbiome of preterm infants is characterized by microbes that commonly dominate when antibiotics are administered (Bifidobacterium spp. deficiency and Enterobacteriaceae, Enterococcus, Staphylococcus prevalence).

In the same year, Rusconi et al. [73] conducted a similar investigation. They analyzed feces collected 1 to 5 days before the development of NEC (9 samples) and 19 controls (32 samples) matched for gestational age and birth weight. They identified 764 metabolites that revealed six different pathways between cases and controls, highlighting significant changes in the components of sphingolipid metabolism in the feces of pre-NEC patients compared to controls, with a decrease in ceramides and an increase in sphingomyelins in cases compared to controls. However, the targeted analysis of samples from 23 cases and 46 controls, although confirming the initial broad observations, highlighted that the metabolites had only 73% classification accuracy using machine learning. Moreover, hierarchical clustering defined a group associated with sphingolipids which contained 60% of cases but only 13% of controls. Nevertheless, this grouping was not associated with any of the clinical and sample variables analyzed. The authors therefore concluded that caution is still needed before using sphingolipids as widely applied predictive biomarkers.

Another stool metabolomics and microbiomics study was conducted by Brehin et al. [58]. From the analysis of the intestinal microbiota, the microbiome and the metabolome of infants with suspected NEC (NEC-1), they detected in the cases a different metabolomic signature compared to healthy infants, with the greatest divergences by the second month of life, in addition to the differences in the microbiota previously discussed. Indeed, in the first 10 days, NEC-1 infants showed a divergent and more homogeneous intestinal microbiota compared to healthy infants but no significant changes in fecal metabolites were observed. Instead, in the second 10 days of life, the fecal metabolome of NEC-1 infants showed significantly lower levels of serine however the greatest separation in terms of the fecal metabolome, with significantly lower levels of ethanol and leucine in NEC-1 infants, was detected in feces collected in the third 10 days of life.

Deianova et al. [78] conducted a prospective multicenter case–control study, analyzing the fecal samples of 31 preterm infants (born at less than 30 weeks of gestation) from 1–3 days before the diagnosis of severe NEC (15 NEC IIIA, 16 NEC IIIB) and control samples with matching gestational and postnatal ages. Preclinical samples from neonates with NEC were characterized by an increase in five essential amino acids (isoleucine, leucine, methionine, phenylalanine and valine) and a decrease in lysine and ethanolamine ratios compared to control samples. A multivariable model based on isoleucine, lysine, ethanolamine, tryptophan and ornithine modestly discriminated cases from controls (AUC 0.67; *p* < 0.001). However, the authors concluded that it is not yet clear whether these alterations reflect specific metabolic changes and may have a role in the development of early biomarkers for NEC.

Finally, Du et al. [79] investigated the intestinal microbiota and the metabolites of tricarboxylic acids (TCA) in the early diagnosis of NEC. 32 preterm infants’ samples were analyzed, of which 16 were NEC and 16 were non-NEC. The study of TCA metabolites, through targeted metabolomic analysis with mass spectrometry, revealed a significant increase in the concentrations of succinate, L-malic acid and oxaloacetate in the NEC group, whereas no significant difference in alpha diversity or beta diversity between the two groups was detected. However, at the phylum level, an increase in *Proteobacteria* and a decrease in *Actinomycetota* in the NEC group has emerged, together with a significant decrease, at the genus level of *Bifidobacterium* and *Lactobacillaceae*.

Results from the fecal metabolome analysis seem to appear less homogeneous. Nevertheless, even in this case, the detection of alterations affecting some amino acids and metabolism (TCA circle) can be highlighted.

Another fairly recent field of investigation for NEC biomarkers is the search for fecal VOCs. In this regard, the first pilot study was conducted in 2009 by Graner et al. [65], which confirmed the possibility of using fecal VOCs to identify infants at risk for NEC. They indeed observed a reduction in fecal VOCs both in the days before and after the diagnosis of NEC. In addition, 4 specific esters present in the controls, specifically, 2-ethylhexyl acetic ester, decanoic acid ethyl ester, dodecanoic acid ethyl ester, and hexa-decanoic acid ethyl ester, were completely absent from 4 days prior to the onset of illness in infants who developed NEC. A few years later, de Meji et al. [68] showed that VOC profiling by e-Nose allowed them to discriminate NEC infants 2–3 days before the onset of clinical symptoms. Discriminant analyses of a larger, prospective, multi-center study [74] including 8 neonatal units, revealed five individual VOCs associated with NEC in at-risk infants (each with an area under the receiver operating characteristics curve of 0.75–0.76) up to 4 days before clinical diagnosis.
**HIGHLIGHT: MAIN METABOLOMIC ALTERATIONS**◆**Urinary metabolome**: alterations in **amino acids composition** and variation in some **osmolytes** (betaine);◆**Blood metabolome**: alterations in **fatty acid metabolism** (acyl-carnitines) and **amino acids**;◆**Fecal metabolome**: **less homogeneous** nevertheless underlines alterations in **some amino acids** and in the TCA circle;◆**Volatile organic compounds analysis**: quantitative and qualitative change in **fecal VOCs**.

## 6. Conclusions

NEC has a complex and multifactorial pathogenesis. In fact, a single cause has not yet been identified but several risk factors are mentioned in the literature, including prematurity and immaturity of the intestine of preterm newborns, which are also characterized by an anomalous intestinal bacterial colonization and an impaired immune response. In addition, there are significant diagnostic difficulties, especially in the initial stages, since the first symptoms are often rather non-specific, resulting in a delay in identifying the pathology. There is therefore a need for an earlier diagnosis through the identification of non-invasive biomarkers capable of allowing timely prediction and effective discrimination of NEC from other intestinal diseases, in order to guarantee immediate intervention that allows for better outcomes.

The main goal of the present review is to give a global overview of the state-of-the-art regarding metabolomics studies of NEC, by exploring the results that emerged from the different biological samples analyzed. In addition, we have tried to emphasize the importance of a more comprehensive approach, including microbiomics and metabolomics in an attempt to overcome some limitations that have emerged in the studies conducted so far. Indeed, the use of “omics” technologies, especially metabolomics and microbiomics, with new culture-independent techniques, have proven to be fundamental synergistic strategies to study the pathophysiology of NEC and, therefore, allow more accurate identification of potential biomarkers. In fact, despite the variability in the microbial profiles of newborns who developed NEC, supporting the absence of a uniform microbial signature for this pathology, some characteristics seem persistent, determining the presence of different types of intestinal communities in preterm infants. Furthermore, it seems that an intestinal microbiome with a high abundance of Bifidobacteria is a protective factor against NEC. 

Therefore, more in-depth metabolomics and microbiomics studies aimed at further elucidating the microbiota–host cross-talk to define the pathogenic contribution of intestinal flora in NEC are needed. Indeed, they could facilitate the detection of the metabolic pathways potentially involved in the pathology by enabling the identification of specific biomarkers. This objective certainly requires the standardization of the techniques currently in use, the possibility of using quantitative measurements and the sharing of data through metabolomic databases to optimize future and broader validation studies that allow the use of the metabolomic fingerprint for diagnostic purposes.

## Figures and Tables

**Table 1:** NEC metabolomics studies.

Authors	Patients	Samples	Technique	Results
Garner et al. [65] 2009	6 NEC and 7 matched controls	Faecal VOCs	SPME GC/MS	↓ VOCs in the days before and after the NEC diagnosis 2-ethylhexyl acetic ester, decanoic acid ethyl ester, dodecanoic acid ethyl ester, and hexadecanoic acid ethyl ester consistently absent from all NEC faecal samples in the 4 days before the onset of the diseases
Morrow et al. [66] (2013)	11 cases vs. 21 controls	Urine	^1^H-NMR	High urinary ratio alanine/histidine provided a good prediction of NEC Alanine and histidine: discriminant metabolites between NEC Bell Stage I and NEC Bell Stage II
Wilcock et al. [67] (2015)	7 NEC vs. 8 preterms without NEC.	Serum	GC-MS	No metabolomics differences in NEC cases after complete enteral nutrition is introduced. ↑ ‘IL-1b before clinical diagnosis
De Meij et al. [68] 2015	13 NEC, 14 matched controls and 31 sepsis	Faecal VOCs	e-Nose	NEC fecal VOCs profiles could be discriminated from controls, from 2–3 days before clinical symptoms onset
Stewart et al. [69] (2015)	6 NEC, 4 LOS vs. 9 controls	Serum	UPLC-MS	No significant protein among the groups
Stewart et al. [70] (2016)	16 preterm infants (6 NEC and 10 matched controls)	Feces	UPLC-MS	Discriminant metabolites involved into C-21 steroid hormones, linoleate, prostaglandines, leucotrienes pathways
Sylvester et al. [71] (2016)	116 NEC vs. 22876 controls	Blood	MS/MS	Acylcarnitines associated at higher risk of NEC
Wandro et al. [72] (2018)	32 VLBW: 3 NEC 8 OS 21 controls	Feces	GC-MS	No discriminant metabolites found
Rusconi et al. [73] (2018)	9 NEC (stage II–III Bell) vs. 19 controls for broad range metabolomics 23 NEC (stage II–III Bell) vs. 46 controls for targeted metabolomics	Feces	UPLC-MS/MS	↑ Sphingomyelins ↓ Ceramides
Thomaidou et al. [15] (2019)	5 NEC stage I, 10 NEC stage II–III vs. 30 controls	Urine	^1^H-NMR HILIC-UHPLC-MS/MS	25 discriminant metabolites in NEC: ↓ alanine, tirosine, asparagine, proline, betaine, citrate, fumarate, riboflavine, polyols ↑ phenylalanine, arginine, pyridoxine
Probert et al. [74] 2019	32 NEC vs. frequency-matched controls without NEC	Faecal VOCs	SPME GC/MS	Change in fecal VOCs between NEC and controls up to 3–4 days before clinical diagnosis
Brehin et al. [58] (2020)	32 preterms: 11 NEC stage I vs. 21 controls	Feces	^1^H-NMR	↓ serine in NEC-1 ↓ ethanol e leucine in NEC-1 in the 2°month of life.
Sinclair et al. [75] (2020)	887 preterms: 73 NEC vs. 814 controlls	Blood	MS-MS	↓ Citrulline/phenylalanine ratio, 3-idrossioleilcarnitine ↑ phenylalanine/tyrosine ratio and octanoilcarnitine/decanoilcarnitine ratio in cases.
Picaud et al. [76] (2021)	18 VLBW: 6 NEC with food intolerance (group 1, NEC), 6 with food intolerance without NEC (group 2, FI) and 6 controls (group 3, GDT)	Urine	^1^H-NMR	Discriminant metabolites: lattate, betaine, myo-inositol, urea, creatinine e N,N-dimetilglicine between LOS NEC and group 3
Thomaidou et al., 2022 [77]	17 NEC vs. 15 LOS vs. 16 controls	Blood	LC-QTOF-MS	L-carnitine discriminant metabolite in NEC and LOS NEC ↓ PC (16:0/0:0) o LysoPC (16:0/0:0), PC (18:1/0:0) o LysoPC (18:1/0:0) ↑ PC (20:4/0:0)
Deianova et al (2022) [78]	31 preterms (<30 weeks) with severe NEC (15 NEC IIIA, 16 NEC IIIB) and 31 controls;	Feces	HPLC	↑ isoleucine, leucine, methionine, phenylalanina and valine ↓ ratios of lisine and ethanolamine in preclinical samples of NEC newborns
Du et al (2023) [79]	16 NEC vs. 16 controls	Feces	MRM UHPLC LC-MS	↑ succinate, L-malic acid and oxalacetate in NEC

Abbreviations: ^1^H-NMR, *proton nuclear magnetic resonance*; GC-MS, gas chromatography–mass spectrometry; UPLC-MS, ultra-performance liquid chromatography-mass spectrometry; HILIC-UHPLC-MS/MS, ultra-high-performance hydrophilic liquid chromatography-tandem mass spectrometry; MS-MS, tandem mass spectrometry; LC-QTOF-MS, liquid chromatography–hybrid quadrupole time-of-flight mass spectrometry; HPLC, high-performance liquid chromatography; MRM, multiple reaction monitoring; UHPLC, ultra high-performance liquid chromatography; LC-MS, liquid chromatography-mass spectrometry; SPME, solid phase microextraction; VOCs, volatile organic compounds; e-Nose, electronic nose, ↓decrease, ↑ increase.

## Data Availability

No new data were created or analyzed in this study. Data sharing is not applicable to this article.
